# Flavour physics as a window to new physics searches

**DOI:** 10.1098/rsta.2023.0088

**Published:** 2024-02-05

**Authors:** Peter Križan

**Affiliations:** ^1^ Faculty of Mathematics and Physics, University of Ljubljana, Jadranska 19, Ljubljana, Slovenia; ^2^ Jožef Stefan Institute, Jamova 39, Ljubljana, Slovenia

**Keywords:** quarks, leptons, Belle II, LHCb, flavour anomalies

## Abstract

Flavour physics is one of the essential elements of the Standard Model (SM). Experimental studies of weak decays of B and D mesons at B factories have, together with the discovery of the Higgs boson at LHC, provided the final confirmation of the validity of the SM. The present generation of precision flavour physics experiments is looking for departures from the SM. We discuss studies of anomalies in b hadron decays, and studies of rare decays, which are a very promising method for searching for new physics.

This article is part of the theme issue ‘The particle-gravity frontier’.

## 1. Introduction: why flavour physics?

The physics of quark flavours is one of the essential elements in our understanding of elementary particles and their interactions. It was crucial in establishing the Standard Model (SM) as the theory of elementary particles and their interactions. Possibly the most prominent examples of its impact are the prediction of the existence of the charm quark based on the unexpectedly low rate of neutral kaon decay to two muons, and the discovery of the unexpectedly large mixing rate in $B-\bar{B}$ transitions [1], which indicated a large top mass years before the top quark was discovered.

At present, we might be in a similar situation. Several hints of departures from SM predictions have recently been reported, such as anomalies in some b hadron decays to lepton pairs as well as the anomaly in the gyro-magnetic ratio of the muon. These anomalies, if confirmed with higher significance, could indeed signal new physics.

The paper is organized as follows. After presenting the motivation and the experimental facilities, we review the present status of studies of several decays of b hadrons with leptons in the final state, some of which show deviations from the SM predictions. We conclude with plans for further research and with an outlook.

## 2. Facilities

The two B factories, the PEP-II electron-positron collider with the BaBar spectrometer, and KEKB with Belle, have to a large degree shaped particle physics in the first decade of this century [2,3]. An essential element of the success of B factories was the excellent performance of the two accelerators, much beyond their design values. The KEKB [4] collider reached a world record peak luminosity of $2.1\times 10^{34}\;{\mathrm{cm}}^{-2}\;s^{-1}$, exceeding the design value by a factor of more than two. The two experiments accumulated data samples corresponding to integrated luminosities of $557\;{\mathrm{fb}}^{-1}$ (BaBar) and $1041\;{\mathrm{fb}}^{-1}$ (Belle). These samples together contain over 1 billion events with a $B\bar{B}$ pair in the final state.

Experimental studies of weak decays of B mesons at B factories have fully established the CKM quark transition matrix as the only source of violation of the particle-antiparticle symmetry (CP) violation in the SM. Following this success, the two experiments searched for the physics beyond the SM in rare decay modes of B and D mesons and τ leptons, in b→s transitions, in the direct CP violation of B mesons, through D meson mixing, and by looking for lepton flavour violating τ decays [2]. In the data samples collected at the Υ(4S) resonance, just above the $B\bar{B}$ production threshold, as well as in samples of Υ(1S), Υ(2S), Υ(3S) and Υ(5S) decays, they also found evidence for hadronic states that do not fit into the standard meson and baryon schemes. A review of the research methods at the two B factories and results of measurements has been collected in a comprehensive book [2].

The present generation of precision flavour physics experiments, LHCb and Belle II, is looking for new physics phenomena in the form of departures from the SM. For this effort, considerably larger data samples are needed. There are two ways to arrive at such a sample, either by upgrading a B factory to a so-called Super B factory, an $e^{+}e^{-}$ collider with two orders of magnitude higher luminosity, or to study b hadrons produced in proton–proton collisions at the LHC.

### (a) Belle II at SuperKEKB

The Belle II detector [5,6] at the SuperKEKB accelerator complex [7] is a super-B factory experiment covering a wide range of exciting physics topics [8]. To achieve the project’s research goals, a substantial increase of the data sample corresponding to an integrated luminosity of $50\;{\mathrm{ab}}^{-1}$ is needed, and for that, the instantaneous luminosity has to reach the ambitious level of $6\times 10^{35}\;{\mathrm{cm}}^{-2}\;s^{-1}$.

In order to reach this goal, which is an increase of the luminosity by a factor of 30, the KEKB accelerator complex required a substantial upgrade [7]. The essential elements in the increase of the luminosity are a reduction of the beam size at the collision point by a factor of 20, from about 1 μm to 50 nm, and an increase of the beam currents by a factor of two compared with the KEKB values. This is known as the ‘nano-beam’ scheme and was invented by P. Raimondi for the Italian super B factory proposal [9]. Compared with KEKB, the two beams collide at an even larger angle of 83 mrad (22 mrad in KEKB); a somewhat lower beam energy asymmetry of 7 GeV (electrons) and 4 GeV (positrons), instead of 8 GeV and 3.5 GeV, was chosen to reduce the beam losses due to Touschek scattering in the lower energy beam [7]. The modifications of the accelerator complex included a new electron injection gun, a new target for positron production, an additional damping ring for the positron beam, a redesign of the lattices of the low energy and high energy rings, a replacement of short dipoles with longer ones (in the low energy ring), the installation of a TiN-coated beam pipe with ante-chambers, modifications of the RF system and a completely redesigned interaction region (IR) [5,7].

Compared with Belle, the Belle II detector has been designed to operate at an accelerator with a 30 times higher luminosity, and thus has to be able to cope with higher event and background rates [5,6]. To maintain the excellent performance of the spectrometer, the critical issue is to mitigate the effects of higher background levels, which lead to an increase in occupancy and radiation damage, as well as to fake hits and pile-up noise in the electromagnetic calorimeter and neutron-induced hits in the muon detection system. Higher event rates required substantial modifications of the trigger scheme, data acquisition system and computing with respect to the preceding experiments. In addition, improved hadron identification was needed, and a hermeticity at least as good as in the original Belle detector was required.

**Figure 1.**
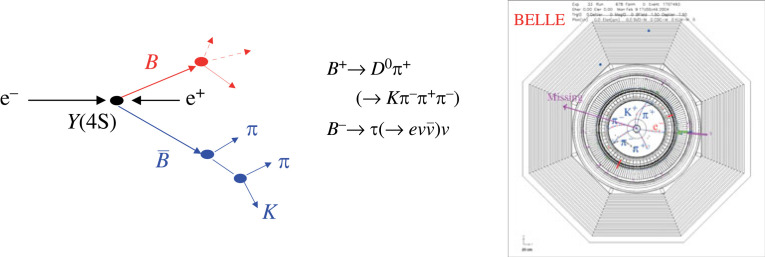
Search for rare processes in B meson decays: if one of the B mesons is fully reconstructed (e.g. in the B→Dπ, D→Kπ decay chain), the remaining particles in the event must be the decay products of the associated B (left); example of a $B^{-}\to \tau^{-}{\bar{\nu }}_{\tau }$ decay, reconstructed with the Belle spectrometer, where the associated B was reconstructed in the decay chain $B^{+}\to {\bar{D}}^{0}\pi^{+}$, ${\bar{D}}^{0}\to K^{+}\pi^{-}\pi^{+}\pi^{-}$ (right).

One of the reasons Belle II needs an excellent hermeticity is a special event analysis method, specific to B factories where exactly two B mesons are produced in a single collision with no additional particles. In this method, one of the B mesons is fully reconstructed in one of a number of exclusive hadronic decay channels such as, e.g. $B\to D^{\left(*\right)}\pi$ (figure 1). The remaining particles in the event must then be the decay products of the associated anti-B. This method, called B-tagging and implemented in Belle II in the Full Event Interpretation (FEI) algorithm [10], is particularly important in searches for rare processes with one or more neutrinos in the final state.

**Figure 2.**
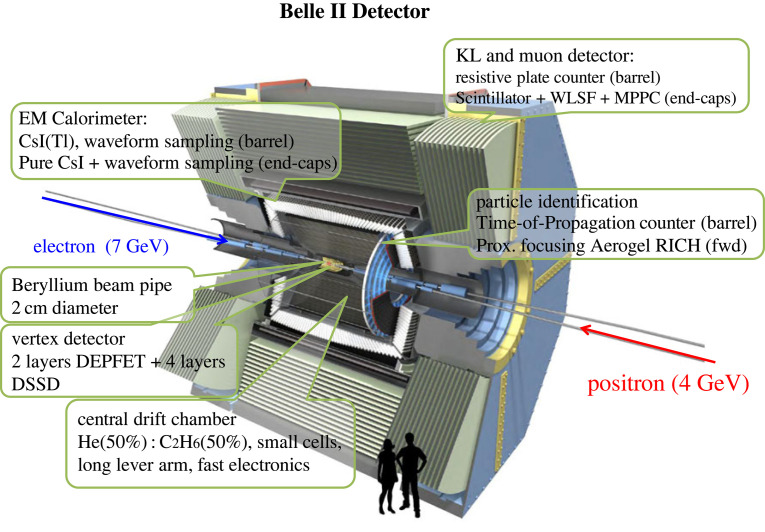
The Belle II spectrometer.

The solutions employed in the Belle II detector [5,6], a spectrometer with a magnetic field of 1.5 T, are displayed in figure 2; they can be summarized as follows. The new vertex detector has six layers around a 10 mm radius Be beam pipe. The first layers at r=14 mm and r=22 mm use pixelated sensors of the DEPFET type (PXD). The remaining four layers at radii of 39, 80, 104 and 135 mm are equipped with double-sided silicon strip sensors. Compared with the Belle vertex detector, the beam pipe and the first two detector layers are closer to the interaction point, and the outermost layer is at a larger radius. As a result, since multiple scattering is an important contribution to the resolution, a significant improvement is expected with respect to Belle in the vertex resolution and in the reconstruction efficiency for $K_{S}^{0}\to \pi^{+}\pi^{-}$ decays with hits in the vertex detector. The central tracking device is again a large volume drift chamber with small drift cells. Compared with Belle, it can extend to a larger radius because of a much thinner PID device in the barrel region. In order to operate at high event rates with increased background levels, the chamber has smaller drift cells than in the Belle case.

Identification of particles is an essential component of most measurements at a B factory. This is particularly important in tagging of the flavour of the associated B meson through the identification of an electron, a muon, or a charged kaon, as well as in the reconstruction of rare B meson decays like B→ππ or Kπ. While electrons are identified in the electromagnetic calorimeter, and muons in an instrumented magnet yoke, hadron identification requires a more complex system. The large kinematic range of hadrons, from a few hundred MeV/c to 4 GeV/c, cannot be covered with a single device; in fact, several detector systems have to be employed to accomplish the goal. Identification of low momentum hadrons is carried out through the measurement of the specific ionization dE/dx. To identify high momentum hadrons, two novel devices were developed, both based on Cherenkov light imaging, a time-of-propagation (TOP) counter in the barrel part [11], and a RICH with an innovative focusing multi-layer aerogel radiator in the forward region of the spectrometer [12].

For the detection of gamma rays and identification of electrons, the excellent original high-resolution Belle electromagnetic calorimeter with CsI(Tl) crystals is employed. In the presence of elevated background levels when compared with the operation in Belle, the relatively long decay time of scintillations in CsI(Tl) crystals considerably increased the overlap of pulses from neighbouring (background) events; to mitigate the resulting large pile-up noise, a new wave-form-sampling read-out electronics system has been installed. In the muon detection system, part of resistive plate chambers was replaced by layers of scintillator strips with wavelength shifting fibres, read out by silicon photomultiplier (SiPMs) to mitigate the problem of large background rates due to neutrons that are mainly produced in electromagnetic showers from background reactions.

### (b) LHCb at LHC

Studies of b hadrons in proton–proton collisions benefit from the very large production cross section (compared with the B meson production cross section in B factories) and from the fact that b hadrons are boosted, and thus travel over considerable distances (of the order of 1 cm) before they decay, in particular, if the detector covers the region around the direction of one of the proton beams. However, because of the low signal-to-background ratios, special care is needed in the design of the trigger and data acquisition systems.

**Figure 3.**
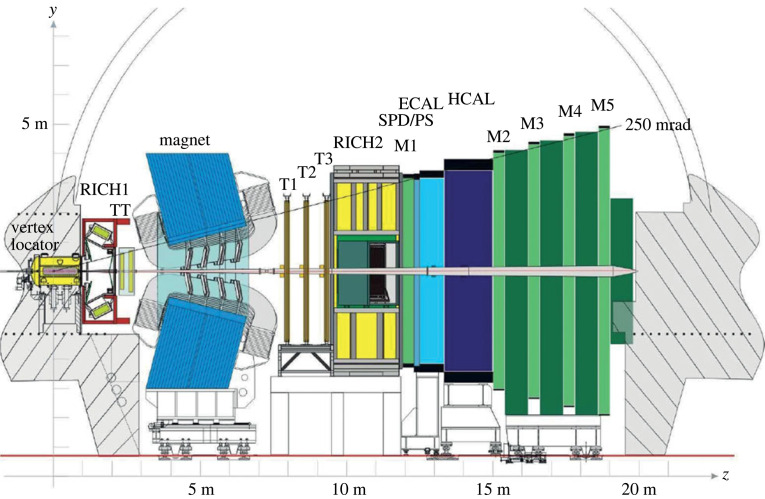
The LHCb spectrometer during the Run 1 and Run 2 data-taking periods.

To accomplish its goals, the LHCb spectrometer [13] shown in figure 3, had to meet the following requirements. First of all, it has to be able to efficiently trigger on hadronic B decays to take advantage from the large b-hadron production rates. It also needs a high-resolution vertex detector for the studies of the time evolution of $B_{s}$ mesons, a reliable tracking system, an excellent PID system for π/K separation, as well as calorimeter and muon systems for electron, hadron and muon identification, especially at the trigger level. Needless to say that all components have to operate at high interaction rates and have to stand very high radiation levels. Finally, the spectrometer needs a high capacity data acquisition system for the large number of read-out channels with a high data throughput, required by the high signal event rates.

The LHCb tracking system has three major parts, a silicon microstrip vertex detector (VELO, Vertex Locator), a silicon microstrip tracker in front of the magnet (TT, Tracker Turicensis) and three tracking stations behind the 4 Tm dipole magnet, with straw tubes for the outer parts (OT, Outer Tracker) and silicon microstrip detectors close to the beam pipe (IT, Inner Tracker).

Similarly as in a B factory, identification of hadrons is an essential element of the experiment. It is needed for tagging of the B meson flavour in CP violation and mixing measurements and for studies of decays to few-body final states like $B^{0}\to \pi^{+}\pi^{-}$ or $B_{s}\to K^{+}K^{-}$. This latter aspect is even more important at LHCb than at Belle II due to the larger range of b hadrons produced, e.g. when studying $B_{s}\to K^{+}K^{-}$ decays one needs to be able to reject efficiently $B^{0}\to K^{+}\pi^{-}$ decays. As a consequence, kaons have to be well separated from pions between 2 GeV/c and 100 GeV/c. To efficiently identify hadrons over such a large kinematic range, the RICH counters of the LHCb experiment [13] employ two gas radiators, $C_{4}F_{10}$ and ${\mathrm{CF}}_{4}$, in two counters, RICH1 and RICH2, as shown in figure 3. Cherenkov photons are detected with a hybrid photon detector (HPD), a vacuum photosensitive device in which photoelectrons are accelerated and focused in the high electric field across a potential difference of about 20 kV, and are subsequently detected in a silicon detector with pixel readout. The two RICH counters have performed extremely well, and have to a large degree contributed to the success of the experiment.

The calorimeter system consists of an electromagnetic shashlik type calorimeter (ECAL) and a hadronic calorimeter (HCAL) with an iron absorber and scintillator tiles. In front of the electromagnetic calorimeter, there is a pre-shower detector to enhance the electron/pion separation, and a scintillator pad detector for the electron discrimination against $\pi^{0}$ mesons. The muon detector employs MWPCs for muon tracking (except in the highest rate region, where triple-GEMs are used).

One of the main components of the LHCb spectrometer is the trigger system. The first level (Level-0) reduces the rate of potentially interesting events from the LHC beam crossing rate of 40 MHz to a rate of 1 MHz with which the entire detector can be read out. Due to their large mass, decays of B mesons often produce particles with large transverse momenta ($p_{T}$) and energies ($E_{T}$), respectively. The Level-0 trigger attempts to reconstruct the highest $E_{T}$ hadron, electron and photon clusters in the calorimeters, and the two highest $p_{T}$ muons in the muon chambers. In order to be able to reduce the event rate from 1 MHz down to 12 kHz, a higher-level trigger (HLT) based on a large computer farm makes use of the full event data. Two important innovations allowed to record data at such a high rate: real time analysis, buffering events selected at Level-0 trigger and in the first step of HLT (HLT1) so that calibration and alignment could be performed and second step HLT (HLT2) decisions made on offline quality reconstruction, and the so-called Turbo stream, recording only the information that is relevant to the selected candidate, rather than all the information in a pp bunch-crossing.

## 3. Studies of anomalies in B meson decays

### (a) Studies of anomalies in b→c and b→u transitions

**Figure 4.**
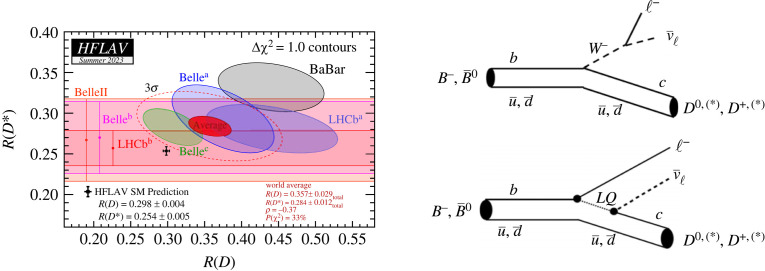
Compilation of measurements of $R(D^{*})$ and R(D) [14] (left); SM prediction is shown as a black point with error bars. Right: Diagrams for the $B\to D^{\left(*\right)}\ell \nu_{\ell }$ transition (with ℓ=e,μ,τ), mediated by the charged SM weak interaction (top), and a non-SM decay process involving a hypothetical leptoquark (bottom).

Recently, some hints of new physics were uncovered in one of the cornerstones of the SM, the so-called Lepton Flavour Universality (LFU) symmetry, i.e. the equality of how the three lepton species, electrons, muons and tau leptons interact via the weak interaction. Within the SM, coupling constants of all charged lepton-neutrino doublets to charged weak bosons are equal. The most interesting hints for a violation of LFU come from the measured deviations of the rates $B\to D^{*}\tau \nu_{\tau }$ and $B\to D\tau \nu_{\tau }$ decays from the SM predictions when compared with $B\to D^{\left(*\right)}\mu \nu_{\mu }$ and $B\to D^{\left(*\right)}e\nu_{e}$ decays. Examples of these decays mediated by the weak interaction are shown in figure 4 (right). To compare experimental data with SM expectations, the ratio of branching fractions $R(D^{\left(*\right)})=BR(B\to D^{\left(*\right)}\tau \nu_{\tau })/BR(B\to D^{\left(*\right)}\ell \nu_{\ell }$), where ℓ=e,μ, is typically used to reduce systematic uncertainties such as those on the experimental efficiency, the CKM matrix elements $|V_{cb}|$ and the form factors. The SM calculations of these ratios assuming LFU have a precision of better than 2% and 1% for $R(D^{*})$ and R(D), respectively [14].

The measured values for $R(D^{*})$ and R(D) are shown in figure 4 (left). The most recent addition to the measurements at BaBar, Belle and LHCb are two measurements by LHCb denoted by LHCb22 [15] and LHCb23 [16], and one by Belle II [17]. The combined measurement, as derived by the HFLAV group [14], is shown as a red ellipse. Taking into account the correlations, the combined difference between the measured and expected values has a significance of slightly above 3σ. Measured discrepancies between the rates of semi-tauonic decays and semi-leptonic decays involving electrons and muons thus hint at a possible violation of LFU as incorporated in the SM. If this anomaly is confirmed with more data, this could point to new physics phenomena, for example, a contribution of a hypothetical leptoquark to the transition (figure 4*, *bottom right).

**Figure 5.**
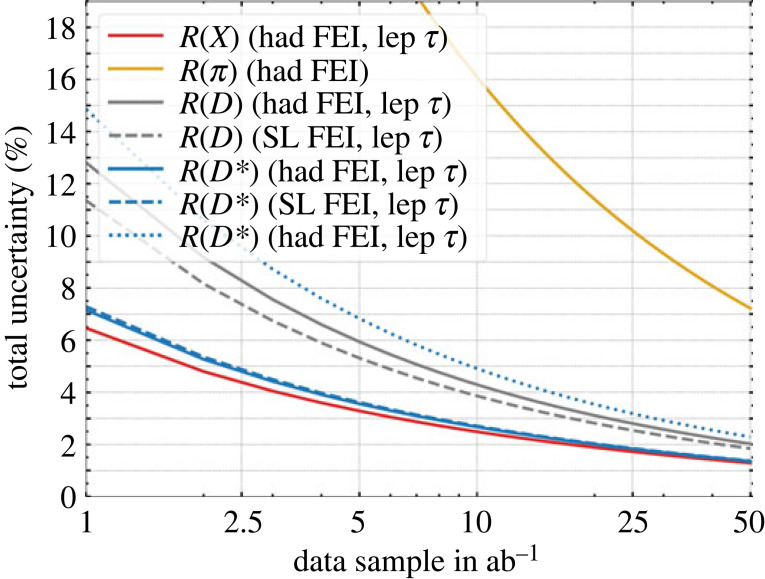
Expected Belle II sensitivity for measurements of various LFU ratios R as a function of luminosity [19]. The FEI acronym refers to the algorithm for the reconstruction of the partner B meson mentioned in §2a.

At Belle II, the first lepton flavour universality test was a comparison of the light lepton branching fractions in $B\to X\ell \nu_{\ell }$ decays with ℓ=e,μ. In the measurement of the ratio $R(X_{e/\mu })=BR(B\to X\mu \nu_{\mu })/BR(B\to Xe\nu_{e})$ they obtained a value of $R(X_{e/\mu })=1.007\pm 0.009\left(\mathrm{stat}\right)\pm 0.019\left(\mathrm{syst}\right)$ [18], which is the most precise lepton-universality test of its kind and agrees with the SM expectation. The first Belle II measurement of $R(D^{*})$ has just been presented [17]; Belle II projections for the measurements of $R(D^{*})$, R(D), R(X), R(π) [19] are summarized in figure 5.

### (b) Searches for new physics in b→s transitions

The b→s transitions have traditionally been an area to look for new physics phenomena. In SM, where flavour-changing neutral currents are forbidden, these transitions are dominated by loop and box diagrams, while new physics in these processes could be due to hypothetical leptoquarks and new particles in loops and boxes.

**Figure 6.**
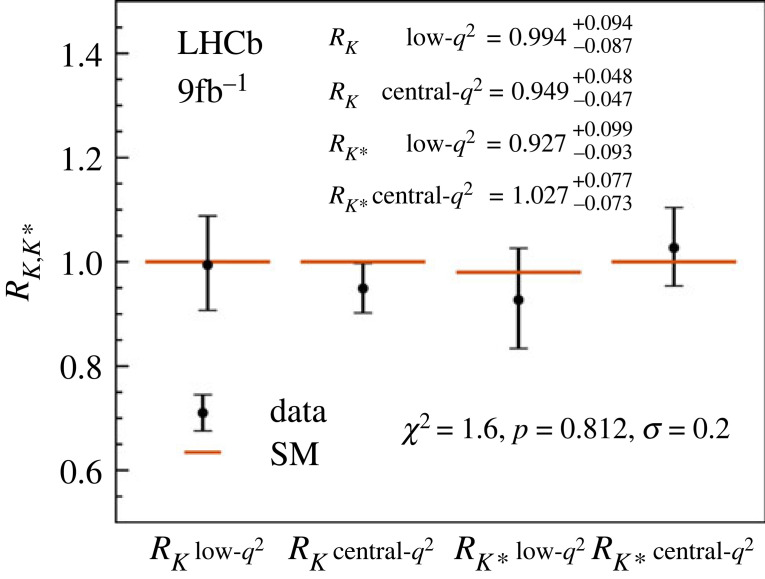
Summary of results for the measurement of $R_{K}$ and $R_{K^{*}}$in two different regions of $q^{2}$ [21].

For some time, the most promising b hadron decay channels in searches for new physics were the decays $B\to K^{\left(*\right)}\mu^{+}\mu^{-}$ and $B\to K^{\left(*\right)}e^{+}e^{-}$, where LHCb saw differences in branching fractions between channels with muon and electron pairs in the final state in the (1.5−3)σ range, while in SM they should be equal. A recent analysis by LHCb [20,21] with improved electron identification and modelling of hadronic backgrounds in the experiment showed, however, that the ratios of branching fractions in two different regions of $q^{2}$ (square of the invariant mass of the two leptons) for either K and $K^{*}$ in the final state, $R_{K^{\left(*\right)}}=BR(B\to K^{\left(*\right)}\mu^{+}\mu^{-})/BR(B\to K^{\left(*\right)}e^{+}e^{-})$, are compatible with 1 (figure 6) and are thus consistent with the SM prediction.

Rare decays of the type b→s still remain, however, a hot topic in particle physics. Among others, searches for new physics are carried out in differential decay rates in $B\to K^{*}\mu^{+}\mu^{-}$ and $B_{s}\to \phi \mu^{+}\mu^{-}$ decays (by LHCb) where further hints for anomalies were seen, searches for rare $B^{\pm }\to K^{\pm }\nu \bar{\nu }$ decays (at Belle II), as well for SM-forbidden lepton-flavour violating decays $B\to K^{\left(*\right)}\tau \ell$ with ℓ=e,μ.

#### (i) Studies of anomalies in $b\to s\ell^{+}\ell^{-}$ transitions

In the decay $B_{s}\to \phi \mu^{+}\mu^{-}$, the differential branching fraction was measured on the full LHCb dataset ($9\;{\mathrm{fb}}^{-1}$) to be $dB/dq^{2}=(2.88\pm 0.22)\times 10^{-8}/({\mathrm{GeV}}^{2}/c^{4})$ for $q^{2}$ between $1.1\;{\mathrm{GeV}}^{2}/c^{4}$ and $6.0\;{\mathrm{GeV}}^{2}/c^{4}$ [22]. This is in agreement with the Run 1 result and corresponds to a 3.6σ deviation from the SM prediction. In the same channel, another tension was observed in the longitudinal polarisation $F_{L}$ of the ϕ meson [23].

**Figure 7.**
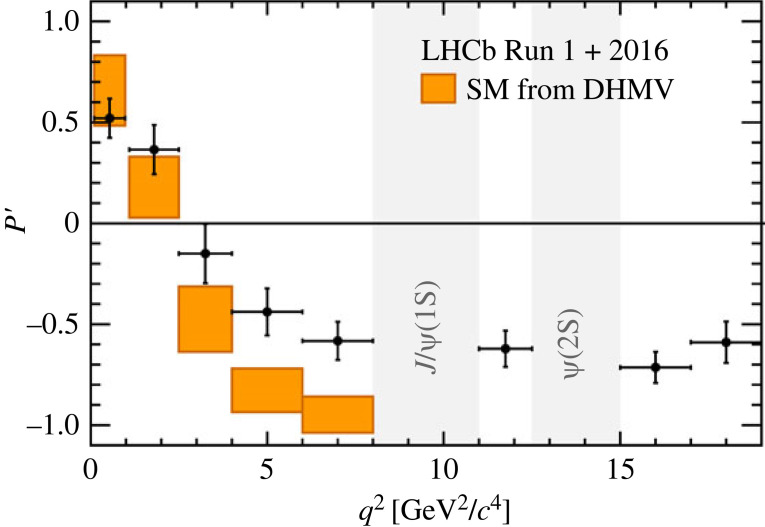
Measurement of the angular observable $P_{5}^{\prime }$ in $q^{2}$ intervals for $B^{0}\to K^{*0}\mu^{+}\mu^{-}$ decays [24].

Angular observables, polarization and asymmetries were studied as a function of $q^{2}$ in the $B^{0}\to K^{*0}\mu^{+}\mu^{-}$ and $B^{+}\to K^{*+}\mu^{+}\mu^{-}$ decays. The measurement of $B^{0}\to K^{*0}\mu^{+}\mu^{-}$ decays [24] showed local tensions of 2.5σ and 2.9σ with SM in the asymmetry parameter $P_{5}^{\prime }$ in $q^{2}$ intervals $[4,6]\;{\mathrm{GeV}}^{2}/c^{4}$ and $[6,8]\;{\mathrm{GeV}}^{2}/c^{4}$ (figure 7).

A global analysis finds a tension of 3.3σ with the SM. These results are consistent with ATLAS, Belle, CMS measurements. In the first LHCb measurement of the decay $B^{+}\to K^{*+}\mu^{+}\mu^{-}$ [25], a local tension with SM of up to 3σ was found in the $P_{2}$ variable (roughly corresponding to the forward-backward asymmetry $A_{FB}$) in the $q^{2}$ interval $[6,8]\;{\mathrm{GeV}}^{2}/c^{4}$.

Fits of the effective field theory Wilson coefficient $Re(C_{9})$ for the three decay channels, $B^{0}\to K^{*0}\mu^{+}\mu^{-}$, $B^{+}\to K^{*+}\mu^{+}\mu^{-}$ and $B_{s}\to \phi \mu^{+}\mu^{-}$, yield negative shifts of $Re(C_{9})$ from the SM preferred value with a 2σ to 3σ significance.

Efforts are underway to measure the third decay channel of this type, $B\to K^{\left(*\right)}\tau^{+}\tau^{-}$, which is clearly even more challenging than the decays to final states with electron or muon pairs because of at least two neutrinos in the final state. An upper limit of $3.1\times 10^{-3}$ at 90% confidence level was set by the Belle collaboration [26] for the decay channel $B\to K^{*0}\tau^{+}\tau^{-}$. More results are expected from LHCb and Belle II.

A related decay, $B_{s}\to \mu^{+}\mu^{-}$, is even rarer because it is additionally helicity-suppressed. The LHCb experiment measured a branching fraction of $(3.09_{-0.43-0.11}^{+0.46+0.15})\times 10^{-9}$ [27] for this decay, while the corresponding result by the CMS experiment amounts to $(2.9_{-0.6}^{+0.7}\pm 0.2)\times 10^{-9}$ [28]. Both results are in agreement with SM predictions.

#### (ii) Search for $B^{\pm }\to K^{\pm }\nu \bar{\nu }$

**Figure 8.**
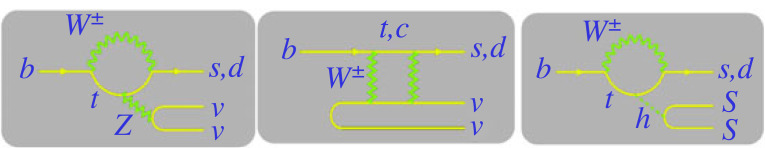
Diagrams contributing to the $B^{\pm }\to K^{\pm }+X$ decays, where X escapes detection; SM loop and box diagrams for $X=\nu \bar{\nu }$ (left and middle), and a diagram for a hypothetical production of pairs of new particles S (right).

**Figure 9.**
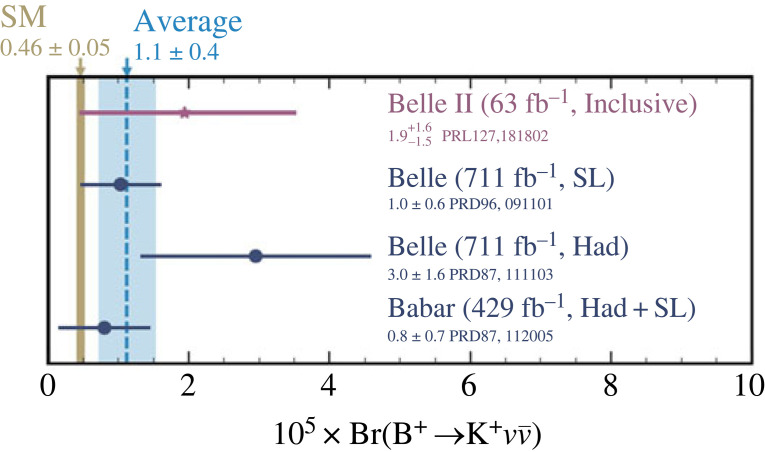
Searches for $B^{\pm }\to K^{\pm }\nu \bar{\nu }$ decays, comparison of results from BaBar, Belle and Belle II; this plot does not include the new Belle II result [30] (see text).

This $B^{\pm }\to K^{\pm }\nu \bar{\nu }$ transition is extremely interesting because its rate could be governed beside the loop and box diagrams in SM (figure 8) with new physics phenomena such as leptoquarks and new particles in loop and box diagrams. Furthermore, instead of the neutrino pair, there could also be a contribution of processes with new particles (e.g. dark matter particles) in the final state. The SM prediction is very clean, $BR(B^{\pm }\to K^{\pm }\nu \bar{\nu })=(4.6\pm 0.5)\times 10^{-6}$ [29]. In experimental studies, one looks for deviations from the expected values, which would give us information on anomalous couplings $C_{L}$ and $C_{R}$ with respect to their SM values (note that the SM value for $C_{R}$ is 0). This flavour-changing neutral current process has been searched for at Belle and BaBar (figure 9) but has not yet been observed. These searches were based on tagged analyses, i.e. by reconstructing the associated produced B meson in semi-leptonic final states with a signal detection efficiency ≈0.2% (at Belle) or in hadronic final states with a signal detection efficiency of about 0.04% (at BaBar). The new approach adopted by Belle II is based on an inclusive tag [31] without an explicit reconstruction of the second B meson, while machine learning (boosted decision trees) are used to exploit distinctive topological features of the decay $B^{\pm }\to K^{\pm }\nu \bar{\nu }$. This method has a much higher signal detection efficiency (4.3%), resulting in increased sensitivity per unit integrated luminosity. As can be seen from figure 9, Belle II reached a similar sensitivity with the new method as did Belle with a data sample 10 times larger.

Further improvements of the analysis on a six times larger data sample have become available in the meantime, resulting in a measured value of $BR(B^{\pm }\to K^{\pm }\nu \bar{\nu })=(2.4\pm 0.7)\times 10^{-5}$ [30]; the significance of the observation is at 3.6 σ, and the result is within 2.8 σ, compatible with the SM value.

#### (iii) Search for the lepton flavour violating b→s transitions

A search for decays of the type $B^{\pm }\to K^{\pm }\tau^{\pm }\ell^{\mp }$ with ℓ=e,μ was carried out using the full Belle data sample. Such lepton flavour violating processes where two leptons of different flavour are produced, are not allowed in the SM; their observation would entail the presence of new mediators, like leptoquarks, allowing for lepton flavour violation. No evidence of these decay types was found; nonetheless, Belle could set the world’s most stringent upper limits on their branching fractions in the $(1-3)\times 10^{-5}$ range at the 90% confidence level [32]. The LHCb experiment set upper limits for the related processes, $B^{0}\to K^{*0}\tau^{+}\mu^{-}$ and $B^{0}\to K^{*0}\tau^{-}\mu^{+}$, both around $1\times 10^{-5}$ at 90% confidence level [33]; they have also set upper limits for $B^{0}\to K^{*0}\mu^{-}e^{+}$ and $B^{0}\to K^{*0}\mu^{+}e^{-}$ decays around $0.7\times 10^{-9}$, and at $1.6\times 10^{-8}$ for $B_{s}\to \phi \mu^{\pm }e^{\mp }$decays [34].

## 4. Outlook and plans

### (a) Outlook for LHCb

The original LHCb spectrometer that was presented in §2b, has been used for data taking in Runs 1 and 2. Recently, a major upgrade of the LHCb spectrometer [35] has been completed. The commissioning of the upgraded detector has been ongoing since the start of Run 3, such that good data-taking is expected in 2024 and 2025, and in particular in Run 4 (2029–2032).

Upgrade I was motivated by the fact that to be able to profit from the excellent performance of the LHC, one has to allow for higher data acquisition rates. Therefore, a completely different triggering strategy was needed, with a fully software-based trigger system. This, however, required that all sub-systems are read out at 40 MHz. As a result, detector systems either needed new read-out electronics or even had to be completely replaced. In addition, detector sub-systems had to be adapted to increased occupancies due to higher luminosity to keep the same excellent performance of the spectrometer.

Silicon microstrip sensors of the vertex detector were replaced by pixel sensors with silicon microchannel cooling and a VELOPIX 40 MHz read-out chip; they were moved closer to the beam, to a distance of 5 mm. While the new upstream tracker is based on silicon strip sensors, the tracking system downstream from the magnet has become uniform, made of scintillating fibre planes, allowing fast pattern recognition. Scintillating fibres are read out by SiPMs outside of charged particle acceptance; to reduce the effects of neutron radiation damage, SiPMs are operated at $-40^{\circ }C$. The main upgrade of the PID system was in the RICH system, where the photo-sensors (HPDs) had to be replaced because they have an embedded read-out electronics chip. The new photosensor is a 64-channel multi-anode PMT, Hamamatsu R11265, read out by a 40 MHz CLARO front-end electronics chip.

A further upgrade, Upgrade II that would fully exploit the LHC facility for flavour physics and beyond, is under preparation. The upgrade is planned for LS4 of LHC, before Run 5 (starting in 2035). The physics case was published in 2018 [36], and the Framework Technical Design Report in autumn 2021 [37]. The mean number of interactions per pp bunch-crossing at the start of each fill will be around 40, making the correct identification of secondary vertices particularly challenging. The increased particle multiplicity and rates will present significant problems for all detectors, as will the increased radiation damage for certain components.

The tracking system will consist of a Vertex Locator (VELO), a silicon-based tracking system upstream of the magnet, and the downstream tracker behind the magnet (Mighty Tracker, MT), split into a Silicon Tracker covering the inner region, and a Scintillating Fiber Tracker (SciFi) covering the outer region. Additional tracking stations will cover the magnet side walls. Hadron identification will again be provided by two RICH detectors, RICH1 and RICH2, upstream and downstream of the magnet; an additional time-of-flight detector (TORCH) will be installed in front of RICH2. Behind RICH2, there will be an electromagnetic calorimeter (ECAL) and 4 muon stations (M2–M5), but no hadron calorimeter. An essential feature of the upgraded spectrometer will be precision timing. This will be required across a number of subsystems, the VELO and most of the particle identification detectors, namely both RICH detectors, ECAL and TORCH. Using a resolution of a few tens of ps per particle will allow charged tracks and photons to be associated with the correct interaction vertex, thereby suppressing combinatorial background.

### (b) Outlook for Belle II

The new SuperKEKB accelerator turned out to present several challenges, with an added operational complexity during the pandemic. It nevertheless reached a record peak luminosity of $4.7\times 10^{34}\;{\mathrm{cm}}^{-2}\;s^{-1}$. The path to reach the next milestone, a luminosity of $2\times 10^{35}\;{\mathrm{cm}}^{-2}\;s^{-1}$, has been identified. There are, however, still large factors to reach the target peak luminosity of $6\times 10^{35}\;{\mathrm{cm}}^{-2}\;s^{-1}$, which may require a further upgrade, for example, a major redesign of the IR.

The path toward higher luminosity is a steep one. Challenges have been encountered in machine performance and stability coming from beam blow-up due to beam-beam effects, shorter than expected beam lifetime, transverse mode coupling instabilities, low machine stability, injector capability and ageing infrastructure. Another set of challenges are backgrounds in the detector [38], with single beam sources (beam-gas and Touschek scattering), luminosity-related (radiative Bhabha and two-photon processes) and injection backgrounds.

Mitigation measures include a consolidation of the accelerator complex and the detector. To improve accelerator performance to the design level, an international task force has been established in 2020 to help with advice and ideas. Many countermeasures are under development.

To consolidate the detector, the complete two layers of the pixel part of the vertex detector have to be installed. Also, a part of the light sensors for one of the particle identification devices, the TOP detector, has to be replaced by more robust devices. These two upgrades are being carried out in the Long Shutdown 1 (LS1) that started end of June 2022 and will be finished by autumn 2023.

A further upgrade of the detector is envisaged to make the detector more robust against backgrounds and improve its performance [39,40]. This would be carried out in the Long Shutdown 2 (LS2), expected to start in 2027 or 2028. LS2 is motivated by a (still to be defined) redesign of the IR, with a replacement of the superconducting final focus quadrupoles. This is a window of opportunity for significant detector upgrades, including a possible replacement of the full vertex detector (the pixel and silicon strip part).

**Figure 10.**
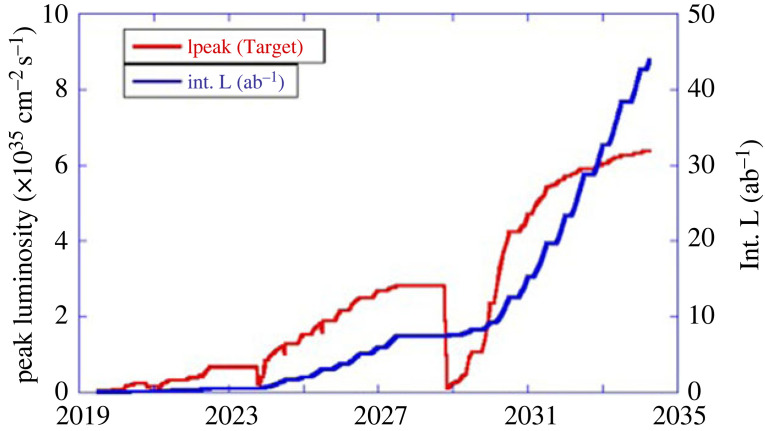
Instantaneous and integrated luminosity projection of SuperKEKB.

The luminosity projection for SuperKEKB is shown in figure 10. The corresponding Belle II physics reach and plans for the next decade and beyond have been updated [39].

An exciting possibility to further expand the experiment’s physics reach would be a polarized electron beam [8]. Such an upgrade would open a new program of precision electroweak physics at the centre-of-mass energy of the Υ(4S), including measurements of $\sin2\theta_{W}$ via left-right asymmetry measurements of electron-positron transitions to pairs of electrons, muons, tau leptons, charm and b-quarks. Other physics enabled with polarized beams includes improved measurements of the properties of the tau lepton and searches for lepton flavour violation as well as topics in hadronic physics. The plan is to reach a 70% polarization with an 80% polarized source. New hardware for the polarization upgrade would include a low-emittance polarized source, spin rotators and a Compton polarimeter to monitor the longitudinal polarization. Beam polarization options at SuperKEKB are under active study.

On an even longer-term (greater than 2032), options are discussed if a significant luminosity increase would become possible. A data sample of $\approx 250\;{\mathrm{ab}}^{-1}$ would be interesting, although it is not clear at this time how to realize such a large further increase in luminosity. A detailed study of the physics case is needed, and technology R&D for an extreme-luminosity detector would have to start soon.

## 5. Summary

Physics of b and c hadrons and τ leptons has contributed substantially to our present understanding of elementary particles and their interactions and continues to be a very hot topic in searches for new physics. Intriguing phenomena seen in recent years make this research area one of the most exciting in particle physics. Many more interesting studies are being carried out at the LHCb, Belle II and BESIII experiments that could not be covered in this review because of lack of space, in particular measurements of the unitarity triangle parameters γ, sin⁡2β, $V_{ub}$, $V_{cb}$, as well as measurements of the CP symmetry violation for D mesons.

B factories have proven to be an excellent tool for flavour physics, contributing a major step in our understanding of flavour, an important part of the SM. The dedicated LHC heavy flavour physics experiment LHCb has been operating extremely well since the start of LHC, and has yielded a plethora of new results. A super B factory has collected its first major dataset at KEK, with the SuperKEKB accelerator and the Belle-II detector.

The LHCb experiment has, in the meantime, finished its Upgrade I and Belle II has entered the super-B-factory regime. For the coming years, we can expect a new, exciting era of discoveries and friendly competition and complementarity of the LHCb and Belle II experiments, as well BESIII, ATLAS and CMS.

## Data Availability

This article has no additional data.
